# Supplementary material for: MetaRibo-Seq measures translation in microbiomes
Source: Nat Commun. 2020 Jun 29;11:3268. doi: 10.1038/s41467-020-17081-z (PMC7324362; doi:10.1038/s41467-020-17081-z)
Supplement: Supplementary file 10 — Supplementary Data 7 [file 41467_2020_17081_MOESM10_ESM.zip › File2/Confidence_High_Taxonomy/227597_out.krona.html]

Javascript must be enabled to view this page.

members
magnitude
magnitudeUnassigned
count
unassigned
taxon
rank

227597\_out

9

2
superkingdom
9

1239
9
phylum

class
9
186801

186802

SRS023971\_contig\_number\_contig-100\_21871.21871SRS893380\_contig\_number\_contig-100\_4486.4487
2
order
9

family
6
541000

2
1263

SRS077194\_contig\_number\_15966SRS1041145\_contig\_number\_contig-100\_8113.8114
genus
6


SRS019445\_contig\_number\_contig-100\_3669.50834
2293189
1
species


SRS062654\_contig\_number\_16052
2293215
species
1


SRS893327\_contig\_number\_3531
2293224
species
1

2293176

SRS055982\_contig\_number\_17454
1
species

family
1
186803

572511
genus
1


SRS024388\_contig\_number\_contig-100\_30485.30485
1805476
species
1
